# Out of Thin Air? Astrobiology and Atmospheric Chemotrophy

**DOI:** 10.1089/ast.2021.0066

**Published:** 2022-02-11

**Authors:** Don A. Cowan, Belinda C. Ferrari, Christopher P. McKay

**Affiliations:** ^1^ Centre for Microbial Ecology and Genomics, Department of Biochemistry, Genetics and Microbiology, University of Pretoria, Pretoria, South Africa.; ^2^ School of Biotechnology and Biomolecular Sciences, Australian Centre for Astrobiology, UNSW Sydney, Randwick, Australia.; ^3^ NASA Ames Research Center, Moffett Field, California, USA.

**Keywords:** Astrobiology, Mars, Titan, Trace gas, Chemotrophy, Redox couple

## Abstract

The emerging understanding of microbial trace gas chemotrophy as a metabolic strategy to support energy and carbon acquisition for microbial survival and growth has significant implications in the search for past, and even extant, life beyond Earth. The use of trace gases, including hydrogen and carbon monoxide as substrates for microbial oxidation, potentially offers a viable strategy with which to support life on planetary bodies that possess a suitable atmospheric composition, such as Mars and Titan. Here, we discuss the current state of knowledge of this process and explore its potential in the field of astrobiological exploration.

## 1. Introduction to Atmospheric Chemotrophy

Atmospheric chemosynthesis, also termed trace gas chemotrophy, is a set of metabolic processes in some microorganisms by which the oxidation of atmospheric trace gases, particularly molecular hydrogen (H_2_), methane (CH_4_), and carbon monoxide (CO), provide the metabolic energy required to drive carbon fixation via one of the most well-known CO_2_ assimilation processes, the Calvin-Benson-Bassham (CBB) cycle (Ji *et al.,* 2017). Hydrogen, carbon monoxide, and methane are present in Earth's atmosphere at low concentrations, typically around 0.6, 0.1, and 1.85 ppm, respectively. A growing number of microbial taxa have been shown to possess the ability to oxidize these gases for energy generation through aerobic respiration, while others use methane and CO as the building blocks for life. Methanotrophy, where atmospheric methane supports both the carbon and energy requirements of select microbial taxa (Lau *et al.,* 2015; Tveit *et al.,* 2019), is probably the best known example of atmospheric chemotrophy. Interestingly, the then-highly speculative concept that H_2_ might act as an energy source that supports microbial survival has existed for more than three decades and was comprehensively reviewed by Morita over two decades ago (Morita, 2000).

In the cold desert soils of Eastern Antarctica, some of which lack photoautotrophs, microbially mediated atmospheric gas chemosynthesis, based specifically on assimilation of CO and H_2_, is thought to support primary production. While it was first assumed that this “overlooked” primary production strategy was a unique and specialized process, there is a growing body of evidence that suggests trace gas assimilation is ubiquitous (Piché-Choquette and Constant, 2019; Bay *et al.,* 2021), and atmospheric chemotrophy may be a generalist metabolic process in desert (and maybe other) soil microbial communities around the globe (Ray *et al.,* 2020; Bay *et al.,* 2021). Given that bacteria with the genetic capacity for trace gas assimilation are thought to literally “live on air,” this discovery has significant implications for early life on Earth and in the search for biosignatures of extant or extinct life on other planets, particularly Mars.

## 2. The Genetics and Enzymology of Atmospheric Chemotrophy

The discovery of atmospheric (H_2_/CO-dependent) chemotrophy as a primary production strategy was based on the application of genome-resolved metagenomics (Ji *et al.,* 2017). This autotrophic process is proposed to involve three core enzymes: hydrogenases, carbon monoxide dehydrogenases, and ribulose bisphosphate carboxylase (RuBisCO). Together, these enzymes act to acquire energy from trace gas oxidation and drive CO_2_ assimilation (through the CBB cycle) to support cellular biosynthetic processes.

Interestingly, both autotrophic and mixotrophic lifestyles have been shown to be supported by trace gas oxidation, with the electrons produced by aerobic respiration used to assimilate CO and CO_2_ via several pathways (King and Weber, 2007; Constant *et al.,* 2010; Greening *et al.,* 2014; Cordero *et al.,* 2019). However, trace gas autotrophy as a primary carbon fixation strategy has only been associated with the CBB cycle (Grostern and Alvarez-Cohen, 2013; Ji *et al.,* 2017; Tveit *et al.,* 2019).

Hydrogenases are metalloenzymes that are involved in the reversible reaction (2H_2_ + O_2_ = 2H_2_O) and include both assimilatory and synthetic enzymes, involved in hydrogen uptake and production, respectively. In soil, approximately 80% of H_2_ uptake is driven by microbially mediated reactions that encompass two distinct processes (Constant *et al.,* 2008). These biphasic processes include classical, low-affinity reactions performed by Knallgas bacteria in the presence of high amounts of substrate (K_M_ H_2_, *ca.* 1000 ppmv) and high-affinity reactions (K_M_ H_2_, < 100 ppmv) that were first demonstrated in *Streptomyces* sp. PCB7 in 2008 (Schuler and Conrad, 1990; Constant *et al.,* 2008). Subsequently, dose-response relationships have been observed in soil communities where elevated H_2_ concentrations resulted in stimulation of low-affinity hydrogenase-expressing Knallgas bacteria and inhibition of high affinity H_2_-oxidizers (Schuler and Conrad, 1990; Piché-Choquette *et al.,* 2018; Piché-Choquette and Constant, 2019). Given the widespread abundance of type 1 [NiFe]-hydrogenases in oligotrophic environments, high-affinity oxidation appears to occur when substrates required for low-affinity hydrogenotrophy are limited, thus providing an alternative and stable energy source for cellular maintenance.

The newly discovered assimilatory type 1h [Ni-Fe]-hydrogenases appear to be uniquely involved in scavenging of atmospheric H_2_ for energy generation (Constant *et al.,* 2010; Greening *et al.,* 2014, 2015, 2016; Liot and Constant, 2016). Along with carbon monoxide dehydrogenases, these enzymes enable the use of H_2_ and CO as electron donors for the aerobic respiratory chain, primarily to support cellular survival, but also in some cases growth (Jordaan *et al.,* 2020; Tveit *et al.,* 2021). To date, assimilatory hydrogenases have been identified in taxonomically diverse species of prokaryotes, with organisms harboring [NiFe]-hydrogenase encoding genes distributed across a significant number of soil taxa, currently spanning 36 bacterial and 6 archaeal phyla (Vignais and Billoud, 2007; Greening *et al.,* 2016).

The oxidation of atmospheric CO, catalyzed by type1 [MoCu] CO-dehydrogenases, also provides energy and carbon for aerobic carboxydotrophic bacteria to assimilate CO and/or CO_2_ via RuBisCO, the primary step of the most well-known carbon fixation pathway, the CBB cycle (King and Weber, 2007). It is thought that atmospheric chemotrophic bacteria utilize a particular lineage of high substrate-affinity RuBisCO (type 1E). This enzyme remains relatively novel: first discovered in the *Mycobacterium* sp. strain JCI DSM 3803 (Park *et al.,* 2009), it has subsequently been linked to the soil Actinobacterium *Pseudonocardia dioxivorans* CB1190 during autotrophic growth (Grostern and Alvarez-Cohen, 2013) and to volcanic cave microbial communities on Mt Erebus, Antarctica (Tebo *et al.,* 2015). It has also been identified in polar soil mesocosms following hydrogen stimulation (Ji *et al.,* 2017; Ray *et al.,* 2020). Given the relatively recent discovery of atmospheric chemotrophy as a major survival and maintenance energy strategy in microbial communities that occupy hyper-oligotrophic soil niches (Leung *et al.,* 2020), pure culture studies are still required to validate the proposed metabolic pathways and enzymes.

## 3. Extant Microbiology and the Ecological Importance of Atmospheric Chemotrophy

The enzymatic oxidation of gaseous hydrogen is a well-established process in terrestrial and marine microbial communities (Conrad, 1999; King, 2003). However, the critical ecological importance of this process was only recently highlighted with the discovery that trace gas chemotrophy is the dominant carbon and energy acquisition process in unique and environmentally extreme Antarctic soils, where microbial photoautotrophy (largely Cyanobacterial) is very limited or totally absent. This specialized process appears to be restricted to the ubiquitous Actinobacteriota and uncultured members within two candidate phyla: *Ca.* Dormibacterota and *Ca.* Eremiobacterota (previously known as AD3 and WPS-2, respectively) (Ji *et al.,* 2017). Both candidate phyla are found in soil ecosystems but are usually rare, being present at abundances of <1% (Coveley *et al.,* 2015; Ji *et al.,* 2021). Surprisingly, however, in Robinson Ridge (East Antarctica) soils where atmospheric chemosynthesis was first identified, these candidate phyla dominated, representing up to 28% of the soil bacterial taxa detected (Ferrari *et al.,* 2016; Ji *et al.,* 2016).

In Antarctic desert soils, this process was implicated in the provision of maintenance energy in the survival of “dormant” microbial populations (Ji *et al.,* 2017). Thermodynamic calculations based on published maintenance energy requirements for dormant cells (Conrad, 1999; Constant *et al.,* 2010) suggest that atmospheric trace gas assimilation rates are compatible with minimum energy needs (Ji *et al.,* 2017). More recently, hydrogenase genes (including those that encode the type 1h high-affinity assimilatory enzymes) have been found to be widespread in hot desert soils from around the world and associated with a wide range of taxa (Jordaan *et al.,* 2020). In addition, key trace gas assimilating genes have been detected in desert soils that span the “three poles” (Ray *et al.,* 2020), and, most recently, rapid trace gas oxidation rates have been quantitated in soils from across Australia (Jordaan *et al.,* 2020; Bay *et al.,* 2021). The suggestion that H_2_/CO trace gas–driven autotrophy is a globally widespread process in soils (Ray *et al.,* 2020; Bay *et al.,* 2021), as found for atmospheric methanotrophy (Tveit *et al.,* 2019), has contradicted earlier suggestions that this microbially mediated strategy was restricted to extreme soil habitats where the capacity for phototrophy was limited (Ji *et al.,* 2017).

Thermodynamic modeling continues to support the hypothesis that the energy derived from trace gas oxidation can theoretically support heterotrophic microbial communities at times of organic-carbon limitation (Bay *et al.,* 2021). Very recently, it has been discovered that the methylotrophic bacterium *Methylocapsa gorgona* MG08 is able to simultaneously oxidize all three common atmospheric trace gases (CH_4_, CO, and H_2_) to support aerobic growth (Tveit *et al.,* 2021).

Covariation between high-affinity H_2_ and CO oxidation rates along a H_2_ gradient, combined with field observations of the key enzymes responsible in a range of terrestrial ecosystems (Piché-Choquette *et al.,* 2018), including volcanic deposits (Lynch *et al.,* 2014) and Antarctic desert soils (Ji *et al.,* 2017), suggests that atmospheric chemotrophs share similar environmental niches. Quantitative phylogenetic data also suggest that these organisms often belong to the rare biosphere, that is, low abundance (<1%) community members (although some members of the Actinobacteriota, the dominant taxa in many soils, harbor the potential for trace gas chemotrophy). However, these taxa appear to provide unique and critical services in oligotrophic soil ecosystems (Woodcroft *et al.,* 2018, Brewer *et al.,* 2019; Ji *et al.,* 2021) and have now been detected in a growing number of oligotrophic and climatically extreme soil ecosystems, including the McMurdo Dry Valleys, the Tibetan plateau (Ray *et al.,* 2020), and Mars analog sites such as the Andean Altiplano saltpans and the Atacama Desert (Lynch *et al.,* 2014).

There is an emerging belief that microbial chemotrophy and photoautotrophy are likely to coexist. One possibility is that trace gas chemotrophy supports carbon and energy acquisition in some ecosystems but supplements alternative sources in others (Ji *et al.,* 2017; Ray *et al.,* 2020). However, it has recently been demonstrated that, within the yet-to-be cultured phylum *Ca.* Eremiobacterota, the order Baltobacterales contains members genetically capable of both anoxygenic photosynthesis and trace gas chemotrophy (Ward *et al.,* 2019; Ji *et al.,* 2021). It seems likely, therefore, that trace gas chemotrophy contributes to the carbon and energy assimilation in all but the most specialized microbial communities, although the relative contributions of trace gas chemotrophy and photoautotrophy in terrestrial and marine habitats have yet to be quantitated.

One of the characteristics of metabolic hydrogen oxidation is *hydrogenesis*; that is, it is water-generating. The ecological relevance of this physiological property is unknown, although it is unlikely to be of any significance in water-sufficient environments. However, in arid and hyperarid soils, which constitute a significant proportion of Earth's land surface area (United Nations Decade Report, 2020), metabolic hydrogenesis may play a significant role (Ortiz *et al.,* 2021; in press). Specialized sublithic and endolithic habitats are the dominant microbial biotopes in many hot and cold deserts (Cary *et al.,* 2010; Cowan *et al.,* 2020), where the microbial communities are typically embedded in extracellular polysaccharide (EPS) matrices (de los Ríos *et al.,* 2014) that both acquire and retain water (through the hygroscopic properties of the matrix: Roberson and Firestone, 1992; Costa *et al.,* 2018). While no quantitative estimates currently exist for *in situ* metabolic hydrogenesis in these specialized niche habitats, we suggest that this process may make a significant contribution to water availability in these communities and, therefore, to water activity and metabolic capacity.

## 4. Planetary Atmospheres and Gas Evolution

By analogy with deserts on Earth, it has been suggested that dry surface soils on Mars could support biological consumption of atmospheric gases, based on the low levels of H_2_, CO, O_2_ in the martian atmosphere (Weiss *et al.,* 2000). Summers *et al.* (2002) modeled the release of CH_4_ from the surface of Mars assuming H_2_ (4H_2_ + CO_2_ = CH_4_ + 2H_2_O) or CO (3CO +2H_2_O = CH_4_ + 2CO_2_) as energy sources. It is generally agreed that moisture availability in the martian subsurface would be the limiting factor for biological uptake (Weiss *et al.,* 2000).

The availability of water on Mars could be ameliorated in two ways: either by implicating saturated salt solutions that depress the freezing point of water or by considering ice as a source of water activity. King (2015) showed that atmospheric CO (at 290 ppm) could be assimilated by halophilic microorganisms in saturated salt solutions, suggesting a possible basis for microbial activity in saturated brine. Brine flows that have been postulated as one explanation for the fluvial features, known as recurring slope lineae, have been frequently observed on Mars (Ojha *et al.,* 2014).

Trainer *et al.* (2019) reported on direct measurements of atmospheric gases over 3 martian years and found mixing ratios of O_2_ to be 1.61 (± 0.09) × 10^−3^ and CO = 5.8 (± 0.8) × 10^−4^. They found that the mixing ratio of O_2_ showed an unexplained seasonal and inter-annual variability, by a factor of 2, ranging from 1.2 × 10^−3^ to 2.2 × 10^−3^. Krasnopolsky and Feldman (2001) reported the H_2_ mixing ratio on Mars of 15 ± 5 ppm in the lower atmosphere. In terms of absolute pressure, this would be 0.009 Pa, given the 610 Pa total pressure on Mars. In comparison, H_2_ in Earth's atmosphere is 0.6 parts per million, equal to 0.06 Pa.

Reports of CH_4_ in the martian atmosphere have varied, but the most detailed surface measurements (Webster *et al.,* 2018) indicate a roughly constant background of 0.4 ppbv with a seasonal variation that ranges from 0.24 to 0.65 ppbv. Webster *et al.* (2018) also reported temporary plumes with concentrations up to about 7 ppb. These values have, however, been called into question and may be due to outgassing by the Curiosity rover, since the Trace Gas Orbiter missions put an upper limit on CH_4_ in the martian atmosphere at around 0.05 ppb (Korablev *et al.,* 2019). Due to its potentially long lifetime in the martian atmosphere (300 years), it has been suggested that CH_4_ may be a useful indicator of biological activity (Summers *et al.,* 2002), as the stable gas would accumulate and diffuse globally.

The atmosphere of Titan, the largest of the saturnian moons, is dominated by N_2_ with CH_4_ at 5.6% and H_2_ at 0.01% (Niemann *et al.,* 2010). In terms of chemical energy, C_2_H_6_ at 10 ppm and C_2_H_2_ at 1 ppm are of “biotic” interest (McKay and Smith, 2005). There is also a rich photochemistry of organic compounds in the upper atmosphere. However, the trace organics (with the exception of C_2_H_4_, at about 1 ppb at the surface) condense in Titan's 70 K tropopause (Fulchignoni *et al.,* 2005) and precipitate to the surface (*e.g.,* Vuitton *et al.,* 2019).

Titan is another interesting example of a location with surfaces that are at least occasionally moistened and in contact with an atmosphere where gases are out of equilibrium. The equatorial regions of Titan are dry but experience occasional rains (Graves *et al.,* 2008; Turtle *et al.,* 2011) that could wet the surface for several days (Williams *et al.,* 2012). The key factor is that, on Titan, the liquid is methane (and ethane), not water. The possibilities of life on Titan in liquid methane and ethane lakes and “rain”-soaked soils at -180°C have recently been reviewed (McKay, 2016). The use of atmospheric gases as an energy source remains an intriguing possibility. Schulze-Makuch and Grinspoon (2005) and McKay and Smith (2005) suggested that H_2_ and C_2_H_2_ could provide a redox couple to support biotic processes. In Titan's lower atmosphere, the mixing ratios of H_2_ and C_2_H_2_ are 0.001 and 10 ppm, respectively, which implies a free energy of reaction of 111 kJ/mol of H_2_ (McKay and Smith, 2005). A comparison of the free energy of this reaction on Titan with the free energy released by methanogenesis on Earth (Table 1) suggests that there is considerably more free energy available from the “Titan” reaction.

**Table 1.** Comparison of Energy Yields for Methanogenesis on Earth and Titan

Location	Reaction	Energy yields under local conditions	Reference
Titan	3H_2_ + C_2_H_2_ = 2CH_4_	111 kJ per mole H_2_	McKay and Smith, 2005
Earth	4H_2_ + CO_2_ = CH_4_ + 2H_2_O	24 kJ per mole H_2_	Sholes *et al.,* 2019

On Titan, the infrequent “rains” may serve the same role as a “water pulse” in deserts on Earth (Leung *et al.,* 2020), which trigger the measurable consumption of H_2_ and C_2_H_2_. The Dragonfly mission to Titan, scheduled for launch in 2027, will survey a range of sites on the surface of Titan. The payload includes a H_2_ sensor to identify gas disequilibrium processes, as possible evidence of life in the liquid methane (Lorenz *et al.,* 2019).

While Earth, Mars, and Titan are the only worlds in the Solar System in which porous and occasionally moist soils are in contact with an atmosphere that contains redox couples, there are aquatic analogs in the outer Solar System. Jupiter's atmosphere contains a zone of clouds with water activity close to unity (Hallsworth *et al.,* 2021a). Cloud water droplets would be in contact with high H_2_ levels and trace organics, which together, as on Titan, would provide a redox couple that could be exploited by biological systems. The saline ocean of Enceladus, one of Saturn's moons, contains low levels of H_2_ and CO_2_ and probably CO (Waite *et al.,* 2009, 2017), potentially available as biotic energy and carbon sources.

## 5. Physicochemical Limits for Life

The outer limits of life (the “biological envelope”) have been a source of fascination for decades, particularly in the fields of extremophilic microbiology and astrobiology (Hallsworth *et al.,* 2021b). A dramatic surge in studies of extremophilic microorganisms, since the 1970s, has led to a nearly stable definition of the biological envelope, where the outer “life” limits of the most dominant abiotic parameters (temperature, pH, radiation) are defined by the most extreme of Earth's extremophilic microorganisms (Harrison *et al.,* 2013; von Hegner, 2020). Significant unknowns remain; however, the longevity of microorganisms under starvation conditions, desiccation (anhydrobiosis), frozen in permafrost (cryobiosis), in the deep subterranean biosphere or in hypersaline inclusions remains an issue of some uncertainty. These unresolved conundrums center on the mechanisms by which microorganisms can survive long periods of starvation limitation combined with one or more of the above states where, in the absence of basal cellular “maintenance” metabolism and repair processes, molecular degradation induced by background ionizing radiation should lead to cell death in much shorter time spans. The implication is, therefore, that viable cells in such specialized long-term survival environments must maintain some degree of metabolic activity capable of driving sufficient energy generation to support cellular maintenance and thus persist.

One of the concerning issues around this proposal has always been the concept that dormant cells are essentially ametabolic, such as in the state of anhydrobiosis (*e.g.,* Keilin, 1959). However, using both traditional (*e.g.,* radiolabeling: Cowan *et al.,* 1979) and modern molecular (*e.g.,* metatranscriptomics: León-Sobrino *et al.,* 2019) methods, researchers have come to perceive that dormant cells retain a low level of metabolic activity and persist. For example, it has recently been observed that some microbial taxa in hyperarid soils continue to express genes encoding nitrogen acquisition enzymes (particularly nitrate reductases: León-Sobrino *et al.,* 2019).

The other issue of concern is that continued metabolism requires a continuous supply of reduced substrate, and long-term utilization of oxidizable organic substrates is a non-equilibrium process; that is, organic substrates in the cellular microenvironment will be depleted. The implication of gas-dependent chemotrophy effectively resolves this dilemma, in that the high diffusability of atmospheric gasses and the low activation energy requirements are likely to continually replenish the reduced substrate supply (Piché-Choquette *et al.,* 2018). We therefore suggest that trace gas chemotrophy, particularly H_2_ assimilation, may play a critically important role in microbial longevity and survival in extremely oligotrophic, cold, and/or desiccated environments (such as might be found in a suitable astrobiological context). Hydrogen is a common, if low, concentration constituent of many planetary atmospheres and, in particular, is generated abiotically by the radiolysis of water in the presence of various minerals (LaVerne and Tonnies, 2003).

## 6. How Atmospheric Chemotrophy Might Support Life on Mars and Other Planetary Bodies

A long-standing conundrum in the search for extant life or for biomarkers of extinct life on exoplanets, particularly Mars, is what metabolic processes putative martian microbes could employ to obtain the energy required for cell maintenance and growth^1^. The consumption of atmospheric H_2_, CO, and CO_2_ by bacterial chemoautotrophs offers a versatile, minimalistic carbon acquisition strategy that offers new possibilities in the search for extraterrestrial life. The genetic determinants of this metabolic strategy have already been detected in Mars analog sites, which include the Andean Altiplano saltpans, the Atacama Desert (Lynch *et al.,* 2014), and mineral soils from both the Antarctic and high Arctic (Ray *et al.,* 2020).

The metabolic pathways for oxidation of CO and H_2_ are considered some of the most ancient evolutionary processes thought to exist on Earth (Sholes *et al.,* 2019), with prebiotic chemistry models predicting that the atmosphere on early Earth was rich in reducing gases (Tian *et al.,* 2005; Piché-Choquette and Constant, 2019; Zahnle *et al.,* 2020). The presence of the trace gases CO and H_2_ in the martian atmosphere (Krasnopolsky and Feldman, 2001; Trainer *et al.,* 2019) represents a potentially untapped source of free energy, much like that on Earth, which suggests that subsurface extant microbes should be capable of using trace gases as energy and carbon sources (Sholes *et al.,* 2019). Using a one-dimensional photochemical model based on the free energy available from trace gases and predicting gas flux at the subsurface, the authors estimate maximum metabolic activity on the martian surface and calculate that approximately 10^27^ cells, equating to 2–40 × 10^−5^ of Earth's biomass, could be supported through chemoautotrophic processes.

In the past, based on the assumption that the martian atmosphere was at equilibrium, it has been argued that extant life (essentially disequilibrium processes) could not be present (Lovelock, 1975, 1988). However, in recent years it has been reported that the martian atmosphere has the second largest thermodynamic disequilibrium after that of Earth, with approximately 136 J/mol of global atmospheric free energy (Krissansen-Totton *et al.,* 2016). This potentially available energy is linked to the redox pairs CO-O_2_, O_2_-CH_4_ and, to a lesser extent, H_2_-O_2_ (Sholes *et al.,* 2019).

**FIG. 1.**
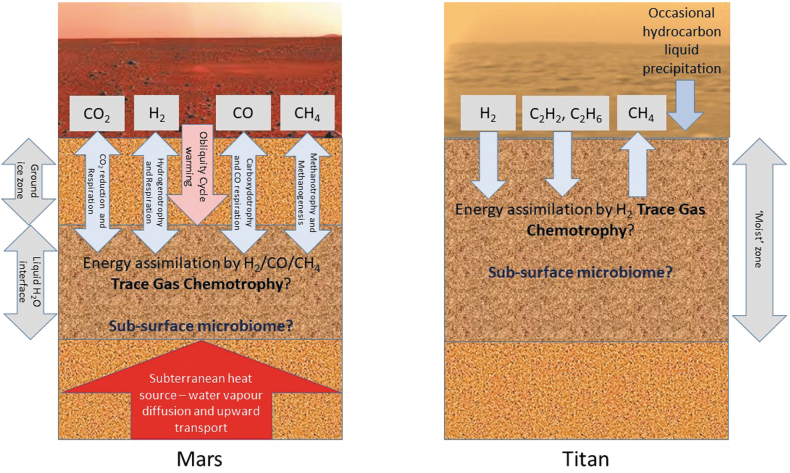
A model for the possible role of trace gas chemotrophy in the survival of soil microbiomes on Mars and Titan.

To some extent, the discovery that trace gas chemotrophy is probably a widespread metabolic process in Earth's terrestrial pan-microbiome offers a new, or at least stronger, paradigm for microbial survival on bodies beyond Earth, particularly Mars and Titan (Fig. 1). In particular, hydrogenotrophy offers exciting opportunities for the long-term energy sustenance of putative subsurface microbiomes. On Mars, the abiotic generation of this high-energy substrate, driven by radioautolysis over geological time, could provide a mechanism for the energetic support of evolutionary processes and for the continued supply of metabolic water, potentially facilitating the selection, adaptation, and survival of martian soil microorganisms on the “dying planet.” On Titan, atmospheric photolysis produces redox couples that could support metabolism in exotic life-forms surviving in liquid hydrocarbons.

## 7. Signatures for Life Based on Signatures of Trace Gas Chemotrophs

The implications of trace gas chemotrophy as a minimalistic mode of carbon fixation are substantial, with the search for life on other planets now prioritizing H_2_ and CO gas (Corenblit *et al.,* 2019; Sholes *et al.,* 2019). While a major research focus is on the development of biosignatures of H_2_ and CO oxidation, there is also the prospect of exploiting redox pairs (CO-O_2_ and H_2_-O_2_) as antibiosignatures, in the case that extant life does not exist (Sholes *et al.,* 2019). Experimental validation of trace gas carbon fixation will undoubtedly aid future predictions for life on exoplanets, particularly H_2_ and CO consumption in the martian atmosphere.

## Abbreviations Used

CBB: Calvin-Benson-Bassham
RuBisCO: ribulose bisphosphate carboxylase

## Notes

^1^

https://mepag.jpl.nasa.gov/meeting/2020-04/whitetopics/WhitePaper_07_Garvin.TraceGas.pdf

## Notes

Associate Editor: Lewis Dartnell
